# Editorial: Dynamics of endocrine system dysfunctions and neuro-ophthalmic manifestations: early diagnostic to therapeutic implications

**DOI:** 10.3389/fendo.2026.1880801

**Published:** 2026-06-12

**Authors:** Dhiraj Kumar, Ahmad Abu Turab Naqvi, Rohan Gupta, Smita Kumari, Rashid Waseem, Md. Imtaiyaz Hassan

**Affiliations:** 1 Section of Retinal Ganglion Cell Biology, Laboratory of Retinal Cell and Molecular Biology, National Eye Institute, National Institutes of Health, Bethesda, MD, United States; 2 Centre for Interdisciplinary Research in Basic Sciences, Jamia Millia Islamia, New Delhi, India; 3 School of Medicine Columbia, University of South Carolina, Columbia, SC, United States; 4 College of Pharmacy, The Ohio State University, Columbus, OH, United States; 5 Laboratory of Biochemistry and Genetics, National Institute of Diabetes and Digestive and Kidney Diseases, National Institutes of Health, Bethesda, MD, United States

**Keywords:** early diagnostics, endocrine dysfunction, neuroendocrinology, neuro-ophthalmic manifestations, precision medicine

## Introduction

The discipline of neuroendocrinology has historically focused on maintaining physiological homeostasis through the feedback loops of the hypothalamic-pituitary-peripheral axes. However, contemporary clinical science increasingly recognizes the dynamic, bidirectional relationship between the endocrine and nervous systems. When endocrine homeostasis is disrupted, the nervous system often acts as both a mediator of the dysfunction and a primary target of its pathological consequences. Among the most sensitive indicators of these systemic disturbances are neuro-ophthalmic manifestations. Because the retina and optic nerve are embryologically derived from the diencephalon, they serve as a highly sensitive, non-invasive “window to the brain,” reflecting the health of both the vascular and neural compartments of the central nervous system 1. This editorial explores the evolving dynamics between systemic endocrinopathies- such as diabetes mellitus and thyroid disorders- and visual health, tracing the continuum from early diagnostic markers to advanced therapeutic implications for the future of integrated patient care 2.

## Pathophysiological dynamics uncovering molecular and immune mechanisms

The interplay between the endocrine and nervous systems creates a complex physiological landscape, where even minor shifts in hormonal signaling can trigger a cascade of neurological symptoms. Recent clinical advances have revealed how systemic endocrine dysfunctions frequently present neuro-ophthalmic disturbances. Manifestations like ocular myopathy and progressive retinal neurodegeneration often serve as the earliest clinical indicators of an underlying endocrinopathy 1. Despite their diagnostic value, these warning signs are frequently overlooked, especially in chronic neurodegenerative conditions such as Alzheimer’s, Parkinson’s, and Huntington’s diseases 3. In these cases, the subtle link between metabolic health and neural decline is missed until irreversible damage occurs. Systemic diseases do not merely cause passive damage to the eye; rather, they trigger active, regulated pathological cascades.

In the context of diabetic retinopathy, pathological dynamics involve a complex crosstalk between neural and vascular degeneration. One of the most compelling themes emerging from this Research Topic is the role of microRNAs (miRNAs) as molecular bridges in retinal neurodegeneration. Recent research highlights the critical role of specific miRNA signatures (e.g., miR-138-5p, miR-144, miR-146a, miR-200a, miR-338-3p, miR-590-3p, and miR-646) to modulate oxidative stress, apoptosis, and inflammatory factors. Notably in driving systemic hyperglycemia-induced retinal neurodegeneration and vascular permeability by directly targeting VEGF, NF-κB, and SIRT1 signaling pathways (Zhao et al., 2024). This molecular understanding reframes the eye from a passive victim of high blood sugar to a site of active dysregulation that could be reversed with targeted therapies.

Similarly, the pathogenesis of Thyroid Eye Disease (TED) reveals dynamic autoimmune interactions within the orbit. The migration of CD34+ fibrocytes to orbital tissues and their expression of immune checkpoints- such as MHC II, B7, and PD-L1 are central to the localized inflammation. When autoantibodies stimulate the TSHR and IGF-1R, T-cell interactions via these checkpoints drive aggressive orbital fibroblast proliferation and hyaluronan synthesis, causing the excessive tissue remodeling characteristic of TED (Shu et al., 2024). Understanding these dynamic immune mechanisms is the beginning toward developing precise interventions.

## Early diagnostics: ocular and peripheral bio-indicators

The transition from reactive treatment to proactive intervention relies entirely on early diagnostics. Ocular and peripheral manifestations often precede severe, irreversible structural damage, offering a critical intervention window. Recent technological advancements in ultra-sensitive detection now allow for the identification of neurological biomarkers at significantly lower concentrations, revolutionizing the potential for non-invasive diagnosis and prognosis 4.

### Advances in objective imaging

In managing thyroid-associated ophthalmopathy (TAO), traditional clinical scoring often fails to capture early, subtle tissue remodeling. Advanced neuroimaging has revolutionized this diagnostic phase. By employing advanced magnetic resonance imaging (MRI) sequences comparing T2 mapping and Dixon fat-suppression techniques, clinicians can objectively quantify fat fraction and water content in extraocular muscles. Crucially, T2 mapping demonstrates superior diagnostic performance (AUC > 0.85) in identifying active inflammatory phases compared to subjective clinical activity scores (Zhang, F. et al., 2024). This shift from subjective clinical assessment to objective radiological quantification allows for precise staging of TAO activity.

### Clinical and physiological biomarkers

Beyond imaging, early diagnostics are being-enhanced by novel clinical metrics. In Graves’ Orbitopathy, standardized Ocular Surface Disease Index (OSDI) evaluations reveal that elevated scores significantly correlate with higher clinical activity and severe proptosis, establishing ocular surface inflammation as a primary, easily measurable marker of active disease (Maglionico et al., 2024).

Concurrently, predicting diabetic peripheral neuropathy (DPN) has advanced through objective biomarkers. Bioelectrical impedance analysis indicates that an elevated extracellular to total body water ratio (ECW/TBW > 0.390) independently predicts DPN, detecting subclinical inflammatory fluid shifts before overt nerve fiber degradation occurs (Chen et al., 2025). Furthermore, elevated serum uric acid (SUA) levels have been established as an independent risk factor for DPN, linking oxidative stress and hyperuricemia-induced endothelial damage directly to peripheral nerve injury (Zhang, X. et al., 2024).

## Therapeutic implications: from symptomatic management to targeted repair

Therapeutically, the frontier of neuroendocrinology is expanding toward molecular targeting and precision medicine. In TED, elucidating the roles of the IGF-1R pathway and immune checkpoints has catalyzed targeted biological therapies, offering a precise alternative to broad immunosuppression (Shu et al., 2024). These treatments effectively reduce inflammatory and fibrotic phases while minimizing systemic side effects. In diabetic neuropathies, miRNA-based interventions have revolutionized the therapeutic frontier that aim towards transcending simple disease stabilization; they hold the potential to actively repair the neural architecture of the retina and reverse endothelial damage (Zhao et al., 2024). These advancements suggest a future where treatment is dictated not just by the disease category, but by the specific molecular profile of the individual patient.

## Future recommendations

The dynamics of endocrine system dysfunctions are profoundly intertwined with neuro-ophthalmic health, establishing the eye as an invaluable bio-indicator of systemic metabolic and autoimmune health. By integrating novel diagnostic markers- ranging from advanced MRI sequences and bioelectrical impedance to molecular miRNA signatures, the medical community can detect systemic diseases in their earliest stages. Furthermore, researchers are increasingly investigating the gut-brain-eye axis to understand how microbiome and systemic inflammation contribute to ocular health. The implementation of artificial intelligence (AI) and machine learning (ML) models is particularly promising for analyzing these multidimensional datasets, helping to seamlessly translate early warning signs into targeted molecular and biological therapies 5. Ultimately, this special issue aims to foster a collaborative environment among endocrinologists, ophthalmologists, and neuroscientists to work together to integrate these findings into routine clinical practice. This multidisciplinary approach is no longer just an academic ideal, but a vital clinical necessity for the modern era of medicine.
